# Human Embryonic Stem Cells and Embryonal Carcinoma Cells Have Overlapping and Distinct Metabolic Signatures

**DOI:** 10.1371/journal.pone.0039896

**Published:** 2012-06-29

**Authors:** Raed Abu Dawud, Kerstin Schreiber, Dietmar Schomburg, James Adjaye

**Affiliations:** 1 Department of Vertebrate Genomics, Max-Planck-Institute for Molecular Genetics, Berlin, Germany; 2 Stem Cell Unit, Anatomy Department, King Saud University, Riyadh, Saudi Arabia; 3 Technische Universität Braunschweig, Institute for Biochemistry, Biotechnology and Bioinformatics, Braunschweig, Germany; 4 Institute for Stem Cell Research and Regenerative Medicine, Faculty of Medicine, Heinrich-Heine University Düsseldorf, Düsseldorf, Germany; Michigan State University, United States of America

## Abstract

While human embryonic stem cells (hESCs) and human embryonal carcinoma cells (hECCs) have been studied extensively at the levels of the genome, transcriptome, proteome and epigenome our knowledge of their corresponding metabolomes is limited. Here, we present the metabolic signatures of hESCs and hESCs obtained by untargeted gas chromatography coupled to mass spectrometry (GC-MS). Whilst some metabolites are common to both cell types, representing the self-renewal and house-keeping signatures, others were either higher (e.g., octadecenoic acid, glycerol-3-phosphate, 4-hydroxyproline) or lower (e.g., glutamic acid, mannitol, malic acid, GABA) in hESCs (H9) compared to hECCs (NTERA2), these represent cell type specific signatures. Further, our combined results of GC-MS and microarray based gene expression profiling of undifferentiated and OCT4-depleted hESCs are consistent with the Warburg effect which is increased glycolysis in embryonic cells and tumor cells in the presence of O_2_ while oxidative phosphorylation (OXPHOS) is impaired or even shut down. RNAi-based *OCT4* knock down mediated differentiation resulted in the activation of the poised OXPHOS machinery by expressing missing key proteins such as *NDUFC1, UQCRB* and *COX*, increase in TCA cycle activity and decreased lactate metabolism. These results shed light on the metabolite layer of pluripotent stem cells and could potentially establish novel metabolic markers of self renewal and pluripotency.

## Introduction

Pluripotent human embryonic stem cells (hESC) find their malignant counterpart in human embryonal carcinoma cells (hECCs) [1], [2], [3]. Pluripotency, defined as the ability to self-renew while retaining the capacity to differentiate into all lineages of the embryo proper make hESCs great candidates for regenerative therapies. Both properties are invaluable for this purpose, because they insure both, a constant supply *in vitro* and, at least equally important, the generation of therapy needed tissue or cells. This is especially thought to hold true for hESCs, because they are truly pluripotent. hECCs are somewhat more restricted, but serve as a useful model for both, carcinogenesis in pluripotent tissues and for studying self renewal [4], [5]. In particular the latter is interesting for the stem cell field, because in contrast to hESCs, hECCs are easier to culture, cheaper, do not require the co-culture with a feeder layer and display more robustness, i.e. almost no spontaneous differentiation. A pre-requisite for clinical applications, however, is to understand not only the developmental and differentiation processes, but also the undifferentiated state of the hESCs. The undifferentiated state has been extensively investigated in hESCs and due to historical reasons even more so in hECCs.

Apart from developmental aspects most of our knowledge on hESCs and hECCs is restricted to morphological, cellular, subcellular, transcriptional and protein levels. Both hESCs and hECCs are round, small, display a high nuclear/cytoplasmic ratio that dramatically decreases upon differentiation, they contain 1 to 3 prominent nucleoli, the chromatin is rather euchromatic than heterochromatic and they lack or display a shortened G1 phase [1], [3], [6]. However, hESCs grow as colonies unlike hECCs which grow as a monolayer. Despite this difference, both cell types express a variety of common markers on the cell surface – alkaline phosphatase, SSEA3, SSEA4, TRA1-60, but they are negative for SSEA1 [1], [3]. They also express other markers like the core transcription factors OCT4, SOX2, NANOG [4], [5], [7], [8] - that are required to reprogram somatic cells [9], [10] - or the signaling molecules like TGFb, NOTCH, WNT [11], [12], [13], [14], [15].

Although many studies have characterized hESCs and hECCs only very few studies investigated the composition of the metabolome of human pluripotent stem cells [16], [17]. This is rather surprising given that the metabolites and receptor-ligand interactions are amongst the first cell sensors to react to environmental changes. On the other hand many metabolites are instable and therefore limited the analysis on single metabolites for a long time. With the advent of new available technologies like GC-MS and LC-MS, however, it is now possible to assess metabolic profiles. The first study to our knowledge was conducted by Cezar and colleagues who investigated the secreted metabolome of three independent hESC lines and hESC derived neural precursors cells (hNPs) using a liquid chromatography (LC)-electrospray ionization (ESI)- time of flight (TOF) mass spectrometry (MS) approach (LC-ESI-TOF MS) [16]. They detected hundreds of unknown metabolites produced and secreted by both cell types and showed that exposure to valproic acid - a histone deacetylase inhibitor - induced significant changes in a subset of metabolites in hESCs compared to the hNPs [16]. A similar study was performed by Yanes et al. [17]. They investigated the metabolome of murine undifferentiated embryonic stem cells (mESCs) and differentiated mESCs into neurons and cardiomyocytes using an untargeted LC-ESI-MS approach and found that a significant proportion of the metabolites of undifferentiated mESCs are unsaturated carbohydrates. The balance shifted to saturated compounds as soon as the cells differentiated. Hereby, it seems as if the redox potential established by the GSH/GSSG ratio, which decreases upon differentiation, and the ascorbic acid levels, which behave reciprocally upon differentiation, are the driving forces [17]. Panopoulos et al. described recently in an untargeted LC-MS approach that somatic cells undergo a metabolic shift upon reprogramming to iPS cells and that this shift is required for this process to take place [18].

Here, we present the metabolic signatures of the hESC line (H9) and their malignant counterpart hECCs (NTERA2 cl.D1) using an optimized protocol for metabolite extraction using a GC-MS approach adapted to day-to-day culture. This study does not only highlight the metabolic signature of human pluripotent stem cells, but also sheds light on their cancer biology.

## Results

### Establishment of a Metabolite Detection Protocol for hESCs and hECCs

In order to successfully analyze and compare the metabolomes of hESCs and hECCs we have modified and optimized the protocol of metabolite extraction described by Bennett et al. 2008 [19]. We adapted it to day-to-day culture conditions of hESCs and hECCs rendering it suitable to analyze the extracts for untargeted metabolome analysis with GC-MS. First, a washing step with sodium chloride in physiological concentrations was introduced to remove media components from the cells before extraction to minimize any potential noise. This is important as the medium used for the cultivation of eukaryotic cells is complex and contains metabolites that are also present in the cells, e.g. amino acids and sugars in high concentrations. Remaining media components attached to the cells after cultivation would interfere with the relative quantification of intracellular metabolites. A second reason for adapting the protocol for day-to-day culture of hESCs is that large scale culture is costly. We started with as high as 1.5×10^7^ hECCs to insure maximal metabolite detection. Cell numbers were then stepwise reduced to 1×10^6^ cells and below. We were able to implement the same protocol for hESCs. From this step onwards the routinely used cell number for metabolite extractions was 1×10^6^ cells per technical replicate.

### Identified Metabolites

**Figure 1:**
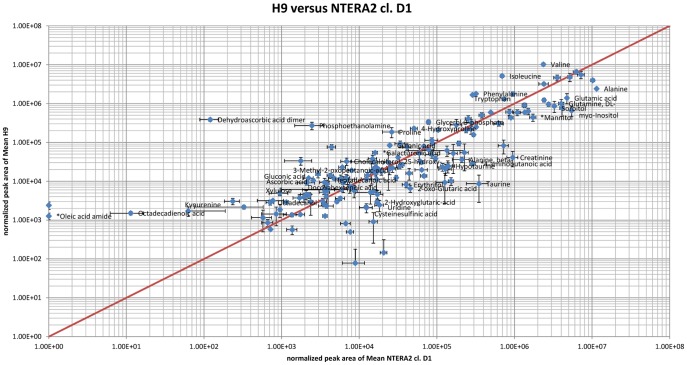
Scatter-plot of normalized peak areas of metabolites detected in hESCs versus hECCs. Only metabolites with more than 3-fold difference are named. Error bars are the standard error.

**Figure 2:**
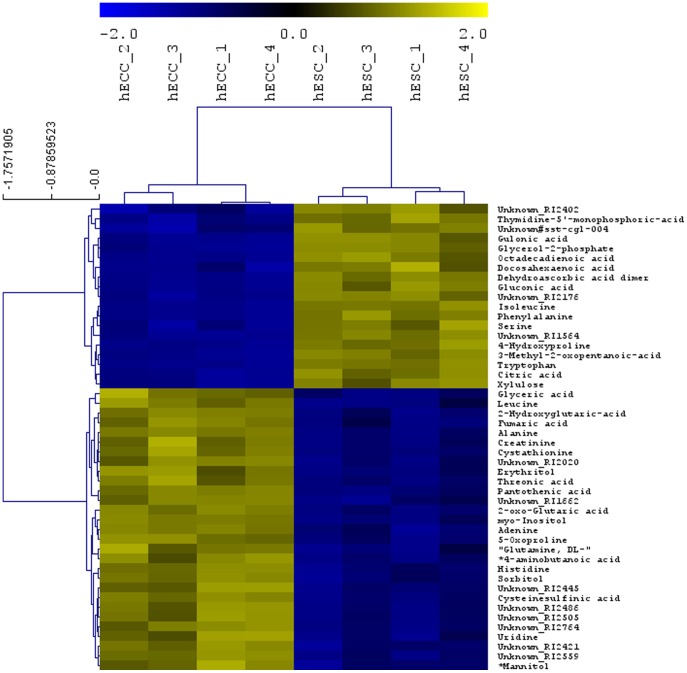
Heat map representing the relative quantities of significantly changed metabolites from polar extracts of hESC versus hECCs. Statistical analysis (T-Test) was done with MeV (p-value <0.05).

The optimised protocol was used for the analysis of the human embryonal carcinoma cell line NTERA2, cl.D1 (hECC) as well as the human embryonic stem cell line H9 (hESC). For both cell types, four biological replicates with two technical replicates each were analyzed. The untargeted GC-MS analysis of the hESCs and hECCs samples resulted in the detection of 161 metabolites of which 57 could not be structurally identified (Tables 1–3 and Table S1). The mean relative standard error after normalization over the 4 biological replicates of the hECCs and hESCs samples was 7.4% and 11.2%, respectively. Figure 1 shows a scatterplot of the normalized peak areas of all substances detected in hESC versus hECC cells. As can be seen in the figure, many metabolites are present in nearly equal amounts, whereas others vary significantly between the two cell types. Taken together, the analysis revealed 68 metabolites that occur in same concentrations in both cell lines (less than 2-fold regulated), 43 metabolites that are higher (more than 2-fold) and 50 that are lower (less than 0.5 fold) concentrated in hESCs compared to hECCs (Table 1–3). All groups contained also unknown metabolites (Table S1). In addition, figure 2 displays those metabolites that differ significantly between hESCs and hECCs according to a T-test performed with MeV with a p-value of <0.05 (adjusted Bonferroni correction) [20], [21].

**Table 1:** Identified metabolites present in hESCs and hECCs.

Ratio hESC/hECC	Metabolite	Info	Significance 1
0.912	**Arginine**	amino acid	
1.868	Asparagine	amino acid	
0.532	Aspartic acid	amino acid	
0.837	Cysteine	amino acid	
1.308	**Glycine**	amino acid	
1.266	Ornithine	amino acid	
1.791	**Serine**	amino acid	**
1.434	Serine, N-acetyl-	amino acid	
0.672	**Tyrosine**	amino acid	
0.514	**Leucine**	amino acid, essential	*
0.707	**Lysine**	amino acid, essential	
0.866	**Methionine**	amino acid, essential	
1.865	**Threonine**	amino acid, essential	
1.007	Trehalose	disaccharide	
1.815	Eicosatetraenoic acid	fatty acid	
1.322	Hexadecanoic acid	fatty acid	
0.928	Hexadecenoic acid	fatty acid	
1.611	Octadecanoic acid	fatty acid	
1.945	Octadecenoic acid	fatty acid	
0.711	Hexadecanol	fatty alcohol	
0.961	Dihydroxyacetone phosphate	Glycolysis	
1.475	Fructose-6-phosphate	Glycolysis	
1.340	**Glucose**	Glycolysis	
1.266	Glucose-1-phosphate	Glycolysis	
1.263	Glucose-6-phosphate	Glycolysis	
0.673	Glyceric acid-3-phosphate	Glycolysis	
1.045	Lactic acid, DL-	Glycolysis	
1.318	*Glycerol	lipids	
0.544	Cholesterol	membrane	
0.874	Adenosine	nucleotides	
0.778	Adenosine-5-monophosphate	nucleotides	
0.806	Guanosine	nucleotides	
0.730	Guanosine-5-monophosphate	nucleotides	
1.068	Thymine	nucleotides	
1.052	Uracil	nucleotides	
1.582	Putrescine	diamine	
1.171	*Galactose	sugar	
1.694	Maltose	sugar	
0.801	Gluconic acid-1,5-lactone	sugar acid	
0.582	Glyceric acid	sugar acid	*
1.773	Xylitol	sugar alcohol	
0.638	Glucose, 1,6-anhydro, beta-	sugar degradation	
1.523	D-Sedoheptulose-7-phosphate	sugar phosphate	
1.144	**Nicotinamide**	vitamine B3	
0.525	**Pantothenic acid**	vitamine B5	**
0.835	myo-Inositol-2-phosphate		
0.937	Phosphoric acid		

Metabolites present in DMEM medium written in bold.

1 Statistical significance was determined by a T-test with adjusted Bonferroni correction in MeV. P-values <0.05 marked as *, p-values <0.01 marked as **.

**Table 2:** Metabolites more than 2-fold higher in hESC compared to hECC cells.

Ratio hESC/hECC	Metabolite	Info	Significance 1
7.080	Proline	amino acid	
7.403	**Isoleucine**	amino acid, essential	**
5.621	**Phenylalanine**	amino acid, essential	*
5.803	**Tryptophan**	amino acid, essential	**
4.343	**Valine**	amino acid, essential	
2.088	2-oxo-Isovaleric acid	valine degradation	
5.436	3-Methyl-2-oxopentanoic-acid	isoleucine degradation	**
27.402	Kynurenine	Tryptophan degradation	
4.298	Docosahexaenoic acid	fatty acid	*
3.319	Heptadecanoic acid	fatty acid	
132.863	Octadecadienoic acid	fatty acid	*
hESC only	*Oleic acid amide	fatty acid amide	
4.010	Octadecanol	fatty alcohol	
2.214	2-Monooleoylglycerol	lipids	
2.469	Glycerol-2-phosphate	lipids	**
4.314	Glycerol-3-phosphate	lipids	
110.622	Phosphoethanolamine	lipids	
4.497	4-Hydroxyproline	membrane	*
2.405	Thymidine-5′-monophosphoric-acid	nucleotides	**
2.552	Spermidine	polyamine	
2.027	Lyxose	sugar	
5.186	Xylulose	sugar	*
3.407	*Galacturonic acid	sugar acid	
4.103	Gluconic acid	sugar acid	*
3.398	Gulonic acid	sugar acid	*
2.015	Citric acid	TCA cycle	**
5.195	Ascorbic acid	vitamine C	
3217.587	Dehydroascorbic acid dimer	vitamine C	**
4.734	Cholecalciferol, 25-hydroxy-	vitamine D3	
2.012	Pyrophosphate		

Metabolites present in DMEM medium written in bold.

1 Statistical significance was determined by a T-test with adjusted Bonferroni correction in MeV. P-values <0.05 marked as *, P-values <0.01 marked as **.

**Table 3:** Metabolites more than 2-fold lower in hESC compared to hECC cells.

Ratio hESC/hECC	Metabolite	Info	Significance 1
0.389	5-Oxoproline	amino acid	**
0.211	Alanine	amino acid	*
0.173	Alanine, beta-	amino acid	
0.294	Glutamic acid	amino acid	
0.415	Glutamic-acid_methylester	amino acid	
0.250	**Glutamine, DL-**	amino acid	*
0.411	**Histidine**	amino acid, essential	**
0.484	Cystathionine	cysteine biosynthesis	*
0.177	Cysteinesulfinic acid	cysteine biosynthesis	**
0.025	Taurine	cysteine, methionine degradation	
0.265	Sorbitol	disaccharide	**
0.367	*Pyruvic acid	Glycolysis	
0.099	*4-aminobutanoic acid	neurotransmitter	*
0.398	Adenine	nucleotides	*
0.134	Uridine	nucleotides	*
0.483	Fructose	sugar	
0.361	Threonic acid	sugar acid	*
0.255	*Mannitol	sugar alcohol	*
0.203	Erythritol	sugar alcohol	*
0.121	**myo-Inositol**	sugar alcohol	*
0.173	2-Hydroxyglutaric-acid	TCA cycle	**
0.145	2-oxo-Glutaric acid	TCA cycle	**
0.489	Fumaric acid	TCA cycle	**
0.349	Malic acid	TCA cycle	
0.422	Succinic acid	TCA cycle	
0.043	Creatinine		*
0.176	Hypotaurine		

Metabolites present in DMEM medium written in bold.

1 Statistical significance was determined by a T-test with adjusted Bonferroni correction in MeV. P-values <0.05 marked as *, P-values <0.01 marked as **.

### Metabolites with Unmodified Concentration in hESCs and hECCs

This group contained several amino acids, intermediates of the glycolysis pathway (glyceric acid-3-phosphate, dihydroxyacetone phosphate, glucose-6-phosphate), some sugars and sugar acids, nucleotides (guanosine, adenosine, 5-AMP, 5-GMP), some fatty acids (hexadecanoic acid, octadecanoic acid), as well as the diamine putrescine and its precursor ornithine (Table 1). Taken together the identified metabolites play a role in energy generation like glycolysis, but also in nucleotide metabolism, protein biosynthesis, lipid metabolism and others. Additionally, several unknown metabolites were found (Table S1). This group of metabolites constitutes the pluripotency signature besides housekeeping metabolites. The two groups of metabolites present in different concentrations in the two different cell lines represent cell type specific differences that contribute to the understanding of the hESCs and hECCs (Table 2–3).

### Metabolites with Higher Concentrations in hESCs Compared to hECCs

The metabolites increased in the hESCs in comparison to the hECCs contained some amino acids and intermediates of amino acid degradation, several fatty acids, especially unsaturated ones (docosahexaenoic acid, octadecadienoic acid), lipid precursors like glycerol-3-phosphate and phosphoethanolamine, and several unidentified compounds whose mass spectra show similarities to sterols (group 1) as well as 4-hydroxyproline (Table 2 and Table S1). Additionally, the levels of ascorbic acid were much higher (5.2 fold) compared to hECCs, as well as the polyamine spermidine (2.6 fold). The metabolites of this group are involved in protein biosynthesis, lipid metabolism and extra-cellular matrix remodeling (4-hydroxyproline).

### Metabolites with Lower Concentration in hESCs Compared to hECCs

This group contained several amino acids (alanine, glutamic acid, glutamine, beta-alanine), the sugar alcohols erythritol and mannitol, several intermediates of the citric acid cycle (malic acid, fumaric acid, 2-oxoglutaric acid and succinic acid), creatinine, the neurotransmitter 4-aminobutanoic acid (GABA) and some unidentified components (Table 3 and Table S1).

### Correlation of the Transcriptome of Undifferentiated and Differentiated hESCs to the Corresponding Metabolome

Microarray based gene expression profiling was performed on undifferentiated hESCs by utilizing our previously published microarray-based data set pertaining to RNAi-based knockdown of OCT4- a major pluripotency maintenance factor - in the human ES line H1 [5], [15]. Indeed, we were able to confirm the regulation of genes and their associated pathways corresponding to the detected metabolites by GC-MS analysis. Examples of these include the intermediates of the glycolysis, the citric acid cycle, fatty acid metabolism, as well as various pathways of amino acid degradation and biosynthesis. However, we were also able to identify additional pathways using the microarray data. For example, we found transcripts encoding for enzymes involved in the pentose phosphate pathway, fructose and mannose metabolism, glutathione metabolism, glycosphingolipid biosynthesis, GPI-anchor biosynthesis, steroid biosynthesis and many others [22].

### Substantiating the Warburg Effect

The Warburg effect describes the feature of tumor cells and embryonic cells to shift their main energy (ATP) source under aerobic conditions from oxidative phosphorylation to glycolysis [23], [24]. With the increased dependency on glycolysis and the regeneration of reduction equivalents like NADPH+, lactate production increases as well. In order to assess whether this is the case in our cell types of interest we analyzed our above mentioned microarray data sets and analyzed the downstream gene regulatory networks [4], [5], [15].

We found several indications like increased glycolysis and reduced oxidative phosphorylation substantiating this hypothesis (Table 4). Essential genes associated with various cytochrome complexes of the oxidative phosphorylation machinery are not expressed in the undifferentiated state and are induced upon OCT4 knockdown mediated differentiation. However, we found the remaining complex oxidative phosphorylation machinery to be expressed with all its protein subunits of the various complexes that are embedded in the mitochondria. The few non-expressed essential subunits were firstly, *NDUFC1* which is an accessory subunit of the NADH dehydrogenase (complex I) that transfers electrons from NADH to the respiratory chain essential for the oxidative phosphorylation [25]. Secondly, *UQCRB* is a gene encoding a protein which is part of the ubiquinol-cytochrome c oxidoreductase complex (complex III) [26]. This complex contains ten nuclear-encoded and one mitochondrial-encoded subunit and is involved in redox-linked proton pumping. The protein binds ubiquinone and contributes to the transfer of electrons when ubiquinone is bound. Mutations in the human gene cause a complex III deficiency resulting in hypoglycaemia and lactic acidosis [27].

**Table 4:** List of metabolite associated pathways in OCT4 depleted hESCs compared to EGFP depleted control cells.

	No. of pathway enriched genes		Difference in gene numbers	
KEGG_Pathway	OCT4 KD	EGFP KD	OCT4 KD	EGFP KD
hsa03040:Spliceosome	114	113	1	
hsa03018:RNA degradation	51	50	1	
hsa00970:Aminoacyl-tRNA biosynthesis	39	38	1	
hsa00240:Pyrimidine metabolism	76	74	2	
hsa00190:Oxidative phosphorylation	98	94	4	
hsa00020:Citrate cycle (TCA cycle)	27	27	0	0
hsa00563:Glycosylphosphatidylinositol(GPI)-anchor biosynthesis	23	22	1	
hsa00310:Lysine degradation	38	34	4	
hsa00280:Valine, leucine and isoleucine degradation	39	36	3	
hsa00520:Amino sugar and nucleotide sugar metabolism	39	38	1	
hsa00480:Glutathione metabolism	37	35	2	
hsa00562:Inositol phosphate metabolism	39	34	5	
hsa00100:Steroid biosynthesis	16	14	2	
hsa00510:N-Glycan biosynthesis	41	35	6	
hsa00230:Purine metabolism	108	98	10	
hsa00030:Pentose phosphate pathway	22	21	1	
hsa00640:Propanoate metabolism	27	25	2	
hsa00270:Cysteine and methionine metabolism	26	26	0	0
hsa00051:Fructose and mannose metabolism	26	26	0	0
hsa01040:Biosynthesis of unsaturated fatty acids	18	17	1	
hsa00100:Steroid biosynthesis	16	14	2	
hsa00450:Selenoamino acid metabolism	20	19	1	
hsa00564:Glycerophospholipid metabolism	47	47	0	0
hsa00533:Keratan sulfate biosynthesis	12	9	3	
hsa00620:Pyruvate metabolism	26	27		1
hsa00900:Terpenoid backbone biosynthesis	13	13	0	0
hsa00010:Glycolysis/Gluconeogenesis	38	38	0	0
hsa04115:p53 signaling pathway	54	50	4	

Shown are the numbers of genes and their difference enriched for the specific pathways.

Thirdly, subunits of the cytochrome c oxidase (COX), the terminal component of the mitochondrial respiratory chain that catalyzes the electron transfer from reduced cytochrome c (complex IV) to oxygen. The mitochondrially-encoded subunits function in electron transfer, and the nuclear-encoded subunits may function in the regulation and assembly of the complex.

## Discussion

Human embryonic stem cells as well as human embryonal carcinoma cells have been heavily investigated at the genomic, transcriptomic, epigenomic and proteomic levels, but metabolome studies have not been extensively employed. To our knowledge, this is the first study to employ an untargeted GC-MS approach to analyze the global metabolome of hESCs and hECCs. This is of importance, because the challenge in the field of hESC research is to find an approach that allows the detection of metabolites from as little starting material as hESCs day-to-day culture can provide. We adjusted our protocol to be as gentle as possible in order to preserve as many metabolites as possible. We removed the medium and replaced it with ice cold washing buffer on ice and then extracted with prechilled (−20°C) extraction solution on ice.

Using this approach our metabolome analysis of hESCs of hECCs revealed three groups of metabolic signatures which will add to the understanding of self renewal in hESCs and hECCs. Firstly, the metabolites present in similar concentrations in both cell types, hESC and hECCs. These metabolites constitute the pluripotency signature besides housekeeping metabolites. Secondly, the metabolites which are present in higher quantities in hESCs compared to hECCs and, thirdly, vice versa. The latter two constitute cell type specific signatures and will contribute to a more detailed understanding of the various types of human pluripotent stem cells.

Both cell types contained metabolites that were present in equal quantities like glycolysis intermediates. These results were verified by the transcriptomic data obtained by microarray based gene expression profiling of undifferentiated hES cells. Taken together, these results substantiate the Warburg effect which is the reliance of embryonic cells and cancer cells on glycolysis rather than oxidative phosphorylation under aerobic conditions. In order to further substantiate these data, we compared gene expression data from undifferentiated cells with OCT4 depleted cells. Indeed, we managed to identify key components of the various essential mitochondrial respiratory chain complexes like *NDUFC1*, *UQCRB* and cytochrome c oxidase, to be upregulated only upon differentiation. This strongly indicates that the oxidative phosphorylation machinery is only fully active in differentiated cells - when they leave the stem cell compartment. However, the majority of the oxidative phosphorylation components are already expressed on mRNA level suggesting that the whole machinery is in a poised like state and ready to go reminding of the epigenetic poised states of simultaneous methylation of active and inactive marks, H3K4 and H3K27 [28], respectively. Regarding the metabolic state our results are in agreement with Prigione et al. 2010 [29] and Panopoulos et al. [18] demonstrating that upon cellular reprogramming, somatic cells shift their energy supply from oxidative phosphorylation to glycolysis.

It is noteworthy that the TCA and TCA cycle intermediates are enriched in hECCs in comparison to the hESCs. This might indicate that the cancerous hECCs could rely less on the Warburg effect than the hESCs. The TCA cycle is upstream and closely linked with the oxidative phosphorylation. The TCA cycle takes place in the mitochondrial matrix and the generated NADH is fed into oxidative phosphorylation taking place in the mitochondrial membranes. In line with this arguement, we found that xylulose, a ketopentose which is present in the cells as xylulose-5-phosphate as an intermediate of the pentose-phosphate pathway, is present in the hES cells in 5-fold excess compared with the hEC cells. Notably, xylulose-5-phosphate can be converted to glyeraldehyde-3-phosphate which can feed into glycolysis. Here, it can be converted to 1,3-bisphosphoglycerate, an energy rich compound that will be used to generate ATP and NADH. Moreover, xylulose-5-phosphate has also a signaling function which leads to the amplification of glycolysis. Further, xylulose-5-phosphate signaling functions extend to glucose induced lipogenisis [30]. Together with 4-fold higher quantities of glycerol-3-phosphate which is a compound of the glycerophospholipids and 110-fold higher levels of phosphoethanolamine which in turn is a precursor of phosphatidylethanolamine, a major component of the membrane lipids and a growth factor in mammalian tumors, it seems plausible to assume that hESCs and hECCs might have different membrane compositions. However, it is possible that this is a consequence of the elevated signaling sphingolipid S1P degradation which is known to be an important pluripotency factor that acts through ERK1/2 activation [31]. Like many metabolites glycerol-3-phosphate is multifunctional and it shuttles the reduction equivalents to the mitochondria, presumably, to prepare the hESCs for differentiation.

The large difference of the 4-hydroxyproline concentration (4.5-fold) higher in hESCs indicated that the composition of the extra-cellular matrices is different between the two investigated cell types. According to this, also proline (7-fold), which is the precursor of 4-hydroxyproline as well as ascorbic acid (5-fold), the cofactor of the 4-hydroxyproline biosynthesis were strongly upregulated in hESCs.

Further, it is noteworthy that various cell membrane components like octadecadienoic acid and some unidentified components similar to sterols (group 1) are present in much higher quantities in hESCs than in hECCs (octadecadienoic acid 132-fold, docosahexaenoic acid 4.3-fold, heptadecanoic acid 3.3-fold, octadecenoic acid 2-fold and various unidentified components similar to sterols around 2-fold). Fatty acids and sterols are amongst other functions incorporated in membranes and influence the fluidity of cell membranes for example. It is known that cholesterol as well as unsaturated fatty acids disrupt the aligned fatty acids of the cell membranes and therefore counteract the viscosity of the cell membranes. It is possible that the low transfection efficiencies of human pluripotent stem cells and in particular of human embryonic stem cells are associated with its special membrane composition. Consequently, it is justified to speculate that the currently available inefficient lipofection reagents for hESCs and hECCs might be improved significantly if they were adjusted in their lipid vesicle composition to the target membranes of these cell types. Many unsaturated fatty acids have been detected in both cell lines, whereby the concentration is higher in hESCs compared to hECCs. The ω-3 fatty acid, octadecadienoic acid, is more than 130-fold higher concentrated in hESCs than in hECCs while eicosatetraenoic acid was only 1.8 fold higher. It has been shown by Yanes et al. that unsaturated compounds are characteristics of embryonic stem cells, enabling the eradication of reactive oxygen species occurring during differentiation [17]. Following Panopoulos et al. it seems that transmethylation, cellular respiration and energy are necessary changes for reprogramming cause they are functionally shown to affect reprogramming [18].

Another metabolite that was higher (27-fold) in hESCs than in hECCs is kynurenine. It is an aromatic non-proteinogenic amino acid that is a product of the degradation of the essential amino acid tryptophan [32]. Kynurenine constitutes a precursor for the NMDA receptor antagonist and displays neuroprotective properties in animal models. Also, it is an intermediate for the synthesis of NAD [32]. Our metabolome data do not indicate which sub-branch of the kynurenine pathway is active. However, the transcriptome data indicate that the levels of *KATI* and *KATIII,* two of three kynurenine amino transferases that convert kynurenine to kynurenic acid which in turn is a broad spectrum antagonist of excitatory amino acid receptor, are almost 2-fold down regulated and 1.7-fold upregulated, respectively, upon differentiation mediated by OCT4 depletion. In addition, L-kynurenine hydrolase (*KYNU*) is 2.4-fold upregulated. KYNU is involved in the kynurenine sub-branch which synthesizes NAD [33]. And therefore, the difference in kynurenine levels could also be reflecting the truly pluripotent properties of hESCs in comparison to the more restricted pluripotential of hECCs.

hECCs have a reduced capacity to differentiate into all embryonic tissues compared to hESCs. Instead, they have sustained/enhanced their proliferative capacity. In agreement with this is the lower abundance of some amino acids in hECCs in our GC-MS analysis that are required for protein biosynthesis. On the other hand the metabolites detected in higher quantities in hECCs in comparison to hESCs are associated with proliferation, for example higher GABA levels. It is known that GABA and its receptors, in particular the subunit GABRB3, maintain the undifferentiated state and correlate in particular with proliferation [34], [35]. We cannot exclude the possibility that the elevated GABA levels are not associated with the bias of hECCs to differentiate towards neurons.

In the stem cell field, Waddington’s model of the epigenetic landscape is widely discussed and accepted. However, increasingly more pieces of evidence indicate that the first differentiation hurdle to commit to differentiation, as many postulate, does not exist - at least *in vivo*. The Waddington model describes (embryonic) development in terms of the “epigenetic landscape” (gene expression/thermodynamically) aiming to reach the lowest energy state (differentiation/death). Arguments against this first differentiation barrier come from various levels, the genetic, epigenetic and the metabolic. First, in 2011, Loh et al. provided convincing arguements that key pluripotency factors, like OCT4, SOX2 and NANOG, are in fact not maintainers of the undifferentiated state, but lineage specifiers [36]. Indeed, the expression of these factors is required for the true pluripotent state and this is also the very reason for their requirement in the generation for iPS cells, but their function is also essential to determine the germ layers and downstream differentiation [36] (references therein), [37]. Secondly, the bivalent chromatin state, in form of simultaneous occurring active and repressive methylation marks of histone 3 lysine 4 and lysine 27, respectively [28], indicates that this poised state keeps the cells prepared to enter the differentiation process without any roadblocks and to remove any silencing mechanisms. Further, we show in this study that glycolysis and the TCA cycle are fully operative and that the oxidative phosphorylation machinery is largely expressed apart from one factor in almost each of the essential complexes of the respiratory chain – in a poised state.

In agreement with this, we found fatty acids susceptible to oxidation. Decreasing GSH/GSSG ratios that alter the redox potential and probably start the oxidizing processes of several substrates lead to differentiation [17]. It seems as if none of these three levels, genetic, epigenetic and metabolic, aim at trapping the cells in the pluripotent state, but almost the opposite. And, indeed, the truly pluripotent cells exist in a very short time window apart from the germ cells when set into the context of the life span of mammals.

We acknowledge that multiple ESC and ECC lines would have been the optimal for our current study, however, we believe our analysis is robust and representative for the following reasons.

First, the international stem cell initiative characterized a large number of distinct human embryonic stem cell lines and showed that they are very similar in their characteristics, for example, the expression of key transcription factors, surface antigens, and other features [38]. The human embryonic stem cell line H9 used in this study is a representative cell line of these features and was included in the mentioned study.

Second, Greber et al. and Andrews et al. previously demonstrated that hESCs and hECCs share fundamental features and that human embryonal carcinoma cells constitute the malignant counterpart of human embryonic stem cells [4], [39], [40]. For example the cellular morphology, the expression of the key factors OCT4/SOX2/NANOG, the expression of surface antigens SSEA4, SSEA3, TRA 1–60, TRA 2–54, TRA 1–80, and many others.

Third, in our previous study, Greber et al. [4] we used RNAi-mediated knockdown of OCT4, SOX2 and NANOG to demonstrate similar effects in three hECC lines, namely NCCIT, 2102Ep and NTERA2. Based on these findings we decided to use the NTERA2 line for our current study.

Human embryonic stem cells (hESCs), in contrast to human embryonal carcinoma cells (hECCs), require besides autocrine bFGF also paracrine bFGF which has been shown for many hESC and hECC cell lines [3], [4], [34], [41]. Therefore, it is essential for hESCs that their culture medium is supplemented with bFGF whereas it is not required for hECCs. Due to this fact it is difficult to formulate a medium which is tailored to the minimal requirements of both cell types simultaneously. However, despite these differences both cell types share reliance on fundamental signaling pathways (e.g. FGF signaling, the Activin/Nodal axis of TGFß signaling and ERK/MAPK signaling) and the core transcription factors OCT4, SOX2 and NANOG to maintain the undifferentiated state and knockdown of these transcription factors in either hESCs or hECCs induces differentiation [4], [7], [36], [37].

### Conclusion

Taken together our combined data clearly illustrate that the analysis of metabolic signatures obtained by the untargeted GC-MS approach is an important step towards the understanding of human pluripotent stem cells and probably constitute only the tip of the ice berg. This and future studies will help to establish novel pluripotency and differentiation markers at the metabolite level and help to increase our meagre understanding of the nature of the pluripotent state.

## Materials and Methods

### Growth of Adherent Cells

The hESC line H9 was cultured in knock-out serum replacement medium that was conditioned with mouse embryonic fibroblasts. The human embryonal carcinoma cells (NTERA2cl. D1,) were grown in DMEM, 10% FBS Serum (Biochrom). Both cell types were cultivated at 37°C in a humidified 5% CO_2_ atmosphere using tissue culture plastic from TPP.

### Metabolome Analysis

The quenching procedure adapted from Bennett et al. [19] was optimised as follows: Cells were grown in 25 cm^2^ tissue culture flasks until the desired time point resulting in about 2−3×10^6^ cells. The cells were washed twice with 5 ml ice-cold 0.9% NaCl solution to remove remaining medium components. The cells were quenched with 1.5 ml extraction solution (80% methanol containing ribitol (2 µg/ml) as a standard for normalization purposes) and the flasks chilled at −20°C for 5 min. The cells were scraped off from the flask, resuspended in the extraction solution and transferred to centrifugation tubes. After centrifugation (4°C, 9,000×g, 3 min), the supernatant was collected and the cell pellet was again resuspended in 0.5 ml extraction solution and centrifuged again (4°C, 9,000×g, 3 min). The supernatants were pooled, mixed and split into 2×1 ml samples, dried under vacuum at room temperature and stored at −20°C until derivatization.

### Sample Derivatization

At first, samples were dried for 30 min under vacuum prior derivatisation. The pellets were derivatized in 40 µl pyridine containing 20 mg/ml methoxyamine hydrochloride at 30°C for 90 min under constant mixing. After addition of 70 µl MSTFA (N-methyl-N-trimethylsilyltrifluoroacetamide), samples were incubated for 30 min at 37°C followed by 2 h at 25°C with constant agitation. The samples were centrifuged at 14,000×g for 5 min and the supernatants were transferred into glass vials for GC-MS analysis. All samples were analyzed within 24 h after derivatization. A retention index marker (n-alcanes ranging from C10…C36 in cyclohexane) was used to convert retention times to retention index.

### Data Acquisition by GC-EI-MS

GC-MS analysis was done on a Thermo GC Ultra coupled to a DSQII mass spectrometer equipped with an AS3000 autosampler (ThermoScientific, Dreieich, Germany). In summary, 1 µl of the derivatized sample was injected in split mode (1∶25) into a PTV injector (ThermoScientific, Dreieich, Germany). After an initial time of 0.2 min at 70°C, temperature was increased to 330°C at a rate of 14°C/s, followed by an additional constant temperature period at 330°C for 5 min. Gas-chromatography was performed over 60 min on a DB-5MS column (30 m×0.25 mm I.D.) (J&W Scientific, Folsom, USA). Helium flow was set to 1 ml/min. After 1 min at 70°C, temperature was ramped to 76°C with 1°C/min and then increased to 310°C with 6°C/min followed by a constant temperature period at 310°C for 10 min. The transfer line temperature was set to 275°C. Ion source temperature was adjusted to 220°C. Full scan mass spectra were acquired from m/z 40…460 with an acquisition rate of 2.5 scans/s. Solvent delay time was 5.15 min. Data acquisition was done using the Xcalibur software (version 1.4, ThermoScientific).

Additionally the samples were analysed using the Leco Pegasus 4D GCxGC-TOFMS in GC-TOF mode (Leco Instrumente, Mönchengladbach, Germany) equipped with a MPS 2XL autosampler (Gerstel, Mühlheim a.d. Ruhr, Germany). 1 µl of the samples was injected in splitless mode into a PTV injector (Gerstel, Mühlheim a.d. Ruhr, Germany). After an initial time of 0.2 min at 70°C the temperature was ramped to 280°C at a rate of 12°C/s, followed by an additional constant temperature period at 280°C for 5 min. Gas-chromatography was performed on a 7890 Agilent GC over 35 min on a Vf-5MS column (30 m×0.25 mm I.D.) (Varian Deutschland, Darmstadt, Germany). Helium flow was set to constant 1 ml/min. After 1 min at 70°C the temperature was increased to 330°C with 10°C/min followed by an additional constant temperature period at 330°C for 8 min. The transfer line was set to 275°C. Ion source temperature was 275°C. Full scan mass spectra were recorded from m/z 45…600 with 20 scans/s. Solvent delay time was 325 s. Data acquisition was done using the ChromaTOF software (version 4.24, Leco).

### Data Processing and Compound Identification

Data analysis was done with Metabolite Detector (version 2.06, [42]) or the ChromaTOF software (version 4.24, Leco). Both software solutions support automatic deconvolution of all mass spectra from a chromatogram and calculate the retention indices based on the retention index marker.

In Metabolite Detector chromatograms obtained from the DSQ II machine were afterwards analyzed in an untargeted batch analysis. Peaks were identified by comparison of retention index and mass spectrum to a user-defined spectra library using a cutoff of 70% identity. Quantification was done by selected 1…3 unique fragment ions for each individual metabolite.

In ChromaTOF, the obtained Leco GC-MS data were analyzed at first by defining a reference chromatogram for hECCs with the maximum number of detected peaks over a signal/noise threshold of 30 and these compounds were added to a reference. Additional compounds from a reference chromatogram for hESCs were added. After that all chromatograms were matched against the reference with a minimum match factor of 700. Compounds annotated by retention index and mass spectra were compared to a user defined spectra library. Selected fragment ions unique for each individual metabolite were used for quantification.

### Statistical Analysis of the Metabolome Data

A first normalization of the metabolome data was performed by dividing the peak area of every detected compound in each sample by the peak area of the respective internal standard ribitol. The individual biological replicates were normalized to the same median after averaging of the technical replicates. Derivates belonging to one substance were summarized before further data evaluation. Afterwards the mean, standard deviation, standard error and relative standard error of the 4 biological samples of the hECC and hESC samples, respectively, were calculated. Significance of concentration changes was determined using the adjusted Bonferroni T-test [20], [21].

### Microarray Data

Total RNA was extracted using the MiniRNeasy Kit according to the manufacturer’s protocol. The quality was checked by Nanodrop analysis (Nanodrop Technologies). 500 ng were used for biotin-labeled cRNA production using a linear amplification kit (Ambion). Hybridizations, washing, Cy3-streptavidin staining, and scanning were performed on the Illumina BeadStation 500 platform (Illumina) according to manufacturer’s instruction. cRNA samples were hybridized onto Illumina human-8 BeadChips version 3. All basic gene expression data analyses were carried out using the BeadStudio software 3.0. Raw data were background-subtracted, normalized using the “rank invariant” algorithm and then filtered for significant expression on the basis of negative control beads. Pathway analyses were determined according to Gene Ontology terms or mapped to KEGG pathways and BIOCARTA pathways with DAVID 2006 [43], [44] using GenBank accession numbers represented by the corresponding chip oligonucleotides as input. Then gene expression arrays (Illumina) for genome-wide expression analysis was carried out. The OCT4 knock down data were taken from [15].

### Statistical Analysis

A groups comparison of two was made using Student’s two-tailed t-test. Data represent the mean of triplicates and are expressed as mean and standard deviation. All statistical analysis was performed using the SPSS 6.0 statistical software program. P values of <0.05 were considered statistically significant.

## Supporting Information

Table S1 **Complete list of detected metabolites in hESCs and hECCs using GC-MS.**
(XLSX)
